# Sensory processing of deep tissue nociception in the rat spinal cord and thalamic ventrobasal complex

**DOI:** 10.14814/phy2.13323

**Published:** 2017-07-18

**Authors:** Shafaq Sikandar, Steven J. West, Stephen B. McMahon, David L. Bennett, Anthony H. Dickenson

**Affiliations:** ^1^ Wolfson Institute of Biomedical Research University College London London United Kingdom; ^2^ Nuffield Department of Clinical Neurosciences John Radcliffe Hospital Oxford United Kingdom; ^3^ Neurorestoration Group Wolfson Wing Hodgkin Building King's College London London United Kingdom; ^4^ Neuroscience, Physiology and Pharmacology University College London London United Kingdom

**Keywords:** Electrophysiology, nociception + lateral spinal nucleus, thalamus

## Abstract

Sensory processing of deep somatic tissue constitutes an important component of the nociceptive system, yet associated central processing pathways remain poorly understood. Here, we provide a novel electrophysiological characterization and immunohistochemical analysis of neural activation in the lateral spinal nucleus (LSN). These neurons show evoked activity to deep, but not cutaneous, stimulation. The evoked responses of neurons in the LSN can be sensitized to somatosensory stimulation following intramuscular hypertonic saline, an acute model of muscle pain, suggesting this is an important spinal relay site for the processing of deep tissue nociceptive inputs. Neurons of the thalamic ventrobasal complex (VBC) mediate both cutaneous and deep tissue sensory processing, but in contrast to the lateral spinal nucleus our electrophysiological studies do not suggest the existence of a subgroup of cells that selectively process deep tissue inputs. The sensitization of polymodal and thermospecific VBC neurons to mechanical somatosensory stimulation following acute muscle stimulation with hypertonic saline suggests differential roles of thalamic subpopulations in mediating cutaneous and deep tissue nociception in pathological states. Overall, our studies at both the spinal (lateral spinal nucleus) and supraspinal (thalamic ventrobasal complex) levels suggest a convergence of cutaneous and deep somatosensory inputs onto spinothalamic pathways, which are unmasked by activation of muscle nociceptive afferents to produce consequent phenotypic alterations in spinal and thalamic neural coding of somatosensory stimulation. A better understanding of the sensory pathways involved in deep tissue nociception, as well as the degree of labeled line and convergent pathways for cutaneous and deep somatosensory inputs, is fundamental to developing targeted analgesic therapies for deep pain syndromes.

## Introduction

Despite the focus of most preclinical and clinical pain studies on cutaneous sensory processing, nonmalignant musculoskeletal pain remains the most common clinical symptom that causes patients to seek medical attention and is a prevalent cause of disability worldwide (Arendt‐Nielsen and Graven‐Nielsen 2011). Musculoskeletal pain can arise from a variety of conditions including trauma, surgery, back pain, fibromyalgia, and other disorders. Nevertheless, studies on nociception in animals and humans most often employ cutaneous stimulation to measure changes in sensory processing using evoked behavioral or neuronal responses as experimental endpoints, and this clearly occludes nociceptive processing from deeper tissues, such as the muscle, fascia, joints, and viscera. Knowledge of the mechanisms involved in deep tissue nociceptive signaling is therefore limited compared to cutaneous pain. Here, we identify central modulatory centers for muscle nociception at the spinal and supraspinal levels.

Little distinction between peripheral and central pathways signaling in deep tissue nociception and cutaneous nociception is known. The perceptual experience of pain arising from deep somatic tissue and from the skin is clearly different; pain from deep tissues is diffuse and difficult to localize, whereas pain arising from cutaneous tissues is typically sharp and easy to localize (Kellgren 1938; Svensson et al. 1997). Importantly, deep tissue pain is typically referred to superficial structures and cutaneous pain is not usually referred (Marchettini et al. 1990). A number of studies have identified differential terminations of muscle and skin afferents in the spinal cord, with cutaneous nociceptors sending dense projections to laminae I, II, and V, and joint and muscle afferents relaying nociceptive information to laminae I and the deeper dorsal horn (Craig and Mense 1983; Mense and Craig 1988). The chemical expression of dorsal root ganglia neurons innervating skin and deep tissue also differs with more peptidergic staining in deep tissue afferents (O'Brien et al. 1989). Top‐down modulation of deep tissue nociception is also less extensively explored compared to cutaneous nociception. However, changes in the balance of descending facilitation and inhibition have been demonstrated in musculoskeletal pain, including fibromyalgia (Ge et al. 2012; Bosma et al. 2016; Potvin and Marchand 2016).

The lateral spinal nucleus (LSN) is located within the dorsolateral funiculus in the rat spinal cord, differing from the superficial dorsal horn by the nature of its neuropil as cell bodies are surrounded by rostrocaudally oriented myelinated axons (Gwyn and Waldron 1969). LSN neurons are thought to receive input from primary afferents via convergent, polysynaptic pathways derived from the dorsal horn, and project through ascending tracts to the brainstem, hypothalamus, and thalamus (Burstein et al. 1987; Harmann et al. 1988; Battaglia and Rustioni 1992; Jiang et al. 1999). The LSN therefore presents a likely key central structure in the processing of deep nociception. However, the nature of its processing has remained enigmatic to date, and thus a key aim of this study is to characterize the nature of LSN processing of somatosensory input.

The thalamus provides a termination point for spinothalamic tract projections and the integration of somatosensory and nociceptive inputs. The ventrobasal complex (VBC) and ventroposterior inferior nucleus form part of the lateral pathway projecting further to S1 and S2 of the somatosensory cortex, and are largely thought to process sensory discriminative components of nociceptive inputs (Harris 1978; Guilbaud et al. 1980; Patel and Dickenson 2016). Nuclei of the medial pathways, including the mediodorsal caudoventral nucleus and the posterior part of ventromedial nucleus, project to the anterior cingulate cortex and insula, and are therefore thought to process the affective and motivational components of nociception (Bushnell and Duncan 1989; Craig et al. 1994; Davis et al. 1999). Phenotypic alterations in coding of neurons of the ventroposterolateral and ventroposteromedial nuclei of the ventrobasal complex to somatic stimulation following tissue injury or spinal disinhibition has been previously reported to demonstrate the sensitization and plasticity of thalamic neurons in pathological conditions (Sherman et al. 1997; Kim et al. 2007; Patel and Dickenson 2016). However, potential changes in the coding of VBC neurons to somatosensory and visceral inputs following muscle trauma remain unknown (Mallart 1968; De Divitiis et al. 1971; Ohara et al. 2004).

Here we describe a novel characterization of LSN neurons in rodents using in vivo extracellular recordings of neuronal activity and Fos activation of LSN neurons in rats. We further characterize LSN innervation through assessment of primary afferent input to this nucleus, and assess neural coding of cutaneous and deep tissue stimulation in the VBC of the rat thalamus in normal conditions and following acute muscle inflammation.

## Methods

### In vivo electrophysiology

Electrophysiology recordings were performed on adult male Sprague Dawley rats weighing 250–300 g. Rats were anesthetized with isofluorane (4%; 66% N_2_O and 33% O_2_) and secured in a stereotaxic frame. Anesthesia was reduced and maintained at 1.5% isoflurane for the remaining duration of the experiment. A laminectomy was performed to expose L3–L5 segments of the spinal cord for single unit extracellular recordings from LSN neurons ventral and lateral to the superficial dorsal horn (ML: 1.0–1.1 mm bilateral from the central vessel at a depth of 590–730 *μ*m). A craniotomy was performed for multiunit recordings from the ventrobasal complex (B: −2.76 to −3.36 mm, ML: 2.5–3.5 mm bilateral, DV: 5–6.5 mm from the dorsal surface). Stereotaxic coordinates were used with reference to the spinal cord and brain atlases (Paxinos and Watson 2005; Watson et al. 2008). Recordings were made using parylene‐coated tungsten electrodes (A‐M Systems, USA). Mechanical and thermal stimuli were applied to the peripheral receptive field of spinal and thalamic neurons on the hind paw glabrous skin (for LSN recordings receptive fields were often limited to the central pad, occluding toes) and the evoked activity of neurons was visualized on an oscilloscope and discriminated on a spike amplitude and waveform basis using a CED 1401 interface coupled to Spike 2 software (Cambridge Electronic Design, UK).

**Figure 1:**
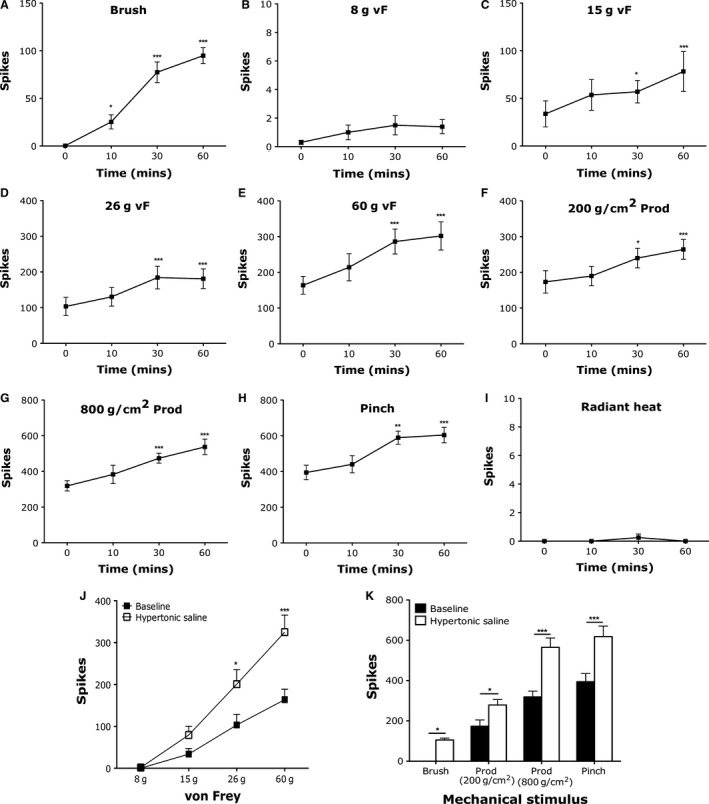
Electrophysiological recordings of LSN neurons demonstrate prolonged sensitization to mechanical stimulation following intramuscular hypertonic saline. Evoked action potentials of LSN neurons at baseline and following hypertonic saline administration to (A) brush, (B) 8 g vF, (C) 15 g vF, (D) 26 g vF, (E) 60 g vF, (F) 200 g/cm^2^ prod, (G) 800 g/cm^2^ prod, (H) pinch, (I) noxious heat (one‐way ANOVA with repeated measures Dunnett's test compared to baseline, *P < 0.05, **P < 0.01, ***P < 0.001). (J) Graded intensity coding to punctate vF stimulation before and maximum change after i.m. hypertonic saline and (K) innocuous brush and noxious prod and pinch mechanical stimulation of the hind paw before and maximum change after i.m. hypertonic saline (*n* = 10 neurons, two‐way ANOVA with repeated measures with Bonferroni post hoc tests, *P < 0.05, ***P < 0.001).

Natural stimuli (vF filaments ranging 2–60 g, noxious prods 200 g/cm^2^ and 800 g/cm^2^ of 1 cm diameter, and thermal water jet ranging 35°C–48°C) were applied in ascending order of intensity to receptive fields for 10 s and the total number of evoked spikes recorded. For LSN recordings, noxious radiant heat stimulation selectively for cutaneous layers was generated by an Nd:YAP laser with a laser beam (wavelength: 1.34 *μ*m; duration: 4 msec; intensity: 4 J, area of beam: 113 mm^2^; ElEn Group). For thalamic recordings we also used colorectal distension of innocuous 20 mmHg and noxious 70 mmHg as described previously (Sikandar and Dickenson 2011; Sikandar et al. 2012). We recorded single units from the LSN (*n *=* *10 cells, one cell per animal), and we used multiunit recordings for the thalamus (*n *=* *33 cells from 10 animals). Following the baseline recordings of evoked neuronal activity, 30 *μ*L of hypertonic saline (10%) was injected into the flexor digitorum brevis and evoked activity to natural stimuli was characterized again at 10, 30, and 60 min following administration. Maximum change from baseline evoked activity was calculated and shown in Figures 1 and 4.

### Immunohistochemistry

#### Fos labeling

Fos immunohistochemistry was performed on adult male Sprague Dawley rats weighing 250–300 g. Rats were anesthetized with isofluorane (4%; 66% N_2_O and 33% O_2_) and were subjected to brush (*n* = 3) or pinch (*n* = 3) stimuli for 10 min. Animals were perfused with saline followed by 4% formaldehyde in 0.1 mol/L phosphate buffer 2 h postsimulation, lumbar spinal cords were dissected, and postfixed for 2 h at 4°C. Tissue was cryoprotected in 30% sucrose, embedded into optimal cutting temperature compound, and stored at −80°C. Cryostat sectioning of transverse tissue sections was performed at a thickness of 20 *μ*m and six sections were collected immediately to Frost Plus slides, allowed to dry at room temperature for approximately 24 h, and stored at −80°C until processing for Fos immunohistochemistry.

Fos immunohistochemistry was performed as follows: After thawing to room temperature and rehydration of tissue sections in PBS, tissue was incubated in 50% ethanol for 30 min to enhance antibody penetration. Tissue was incubated in primary antibody solution (c‐Fos antibody, Santa Cruz, 1:5000; biotinylated IB4, Sigma L2140, 1:100) for 3 days at room temperature in PBS containing 0.3% Triton‐X and 0.1% sodium azide (PBSTxAz). Tissue was washed thoroughly in PBSTxAz followed by incubation in secondary antibody solution (Alexa 546 anti‐Fos, Invitrogen, 1:500; pacific blue–streptavidin, Invitrogen, 1:100) plus NeuroTrace (Invitrogen, 1:100) in PBSTxAz overnight at room temperature. Tissue was washed thoroughly in PBSTxAz, mounted in Vectashield, and imaged on an LSM 700 confocal microscope.

To set the image acquisition settings for imaging c‐Fos labeling, laser, gain, and digital offset settings were adjusted to observe c‐Fos labeling in the ipsilateral L4 dorsal horn of animals subjected to pinch stimuli. These settings were then used for all image acquisition of c‐Fos labeling. All imaging was performed blinded to treatment. Each tissue section was first observed only with the Nissl substance fluorescent stain, and Z stacks of each lateral spinal nucleus were set. Images of NeuroTrace, IB4, and c‐Fos labeling were acquired with identical image acquisition settings. Post image acquisition, all LSN images were blindly labeled and the number of cells in the LSN of each image and number of c‐Fos‐positive cells were quantified.

#### Advillin‐eGFP transgenic mouse immunohistochemistry

We used the Advillin‐eGFP transgenic mouse (Tg‐Avil, GENSAT) to measure primary afferent innervation of the LSN, where GFP expression is under the control of the promotor for the sensory neuron marker Advillin. Hemizygous animals (*n* = 4) were deeply anesthetized with isoflurane and were transcardially perfused with saline followed by 4% formaldehyde in 0.1 mol/L phosphate buffer and lumbar spinal cords dissected. Tissue was postfixed overnight at 4°C, washed in PBS, cryoprotected in 30% sucrose for 24 h, and embedded into OCT for cryosectioning. Lumbar spinal cord sections were cut transverse at 30 *μ*m thickness and mounted to Frost Plus slides, allowed to air dry, and stored at −80°C until processed for GFP histochemistry.

Tissue sections were thawed to room temperature and rehydrated in PBS for 15 min, followed by a 30‐min incubation in 50% ethanol. Sections were then incubated in primary antibodies (goat anti‐GFP 1:200 [Frontier Institute], rabbit anti‐NeuN 1:500 [Abcam], and IB4 conjugated to biotin 1:100 [L2140, Sigma]) in PBSTxAz for 24 h. After washing, the sections were incubated in secondary antibodies (anti‐rabbit 1:500, anti‐goat 1:500, and streptavidin 1:100, conjugated to Cy3, Alexa 488, and pacific blue, respectively). After a final wash step, tissue was mounted in Vectashield (H‐1000, Vector Laboratories) and visualized with a Zeiss LSM 700 confocal microscope.

Tile scan images of 10–12 sections were taken per animal of the spinal cord section. Higher resolution Z stack images throughout the entire depth of the tissue sections were then taken of both the left and right LSN on each spinal cord section to assess EGFP innervation of the LSN. These images were subject to a maximum intensity projection in ImageJ, and primary afferent innervation was then scored for each image, to determine the proportion of LSN samples which showed any arborization of EGFP into the LSN. This was assessed by determining if any GFP‐positive axons extended from the dorsal horn gray matter (identified with the aid of IB4 labeling) into and around the LSN (identified with the aid of NeuN labeling). The number of LSN samples which received such innervation was reported as a percentage of total sections analyzed (no statistical testing was performed on these data).

### Statistical analysis

All data were analyzed using Prism 6 software. Evoked firing of LSN and thalamic neurons before and after hypertonic saline administration was analyzed using a two‐way repeated measures ANOVA with Bonferroni post hoc tests. To determine the number of c‐Fos‐positive cells in the LSN, pinch and brush groups in the ipsilateral and contralateral LSN were compared using a two‐way repeated measures ANOVA with Bonferroni post hoc tests. All data are shown as mean ± SEM and statistical significance set at *P* < 0.05.

## Results

### Electrophysiological and immunohistochemical evidence indicating the role of lateral spinal nucleus in mediating deep tissue nociception

Electrophysiological recordings of evoked activity of LSN neurons to mechanical and thermal stimulation demonstrate a selective response of the spinal nucleus to deep tissue stimulation (Fig. 1). Noxious von Frey hairs, noxious prods, and pinch are all stimuli depressing cutaneous tissue sufficiently to stimulate muscle nociceptors of the hind paw, and these all elicited firing of LSN neurons. By contrast, innocuous dynamic (brush; Fig. 1A) and low‐intensity punctate mechanical von Frey stimulation (8 g; Fig. 1B) that only activates cutaneous sensory afferents failed to activate LSN neurons. Higher intensity von Frey stimulation (15, 26, and 60 g; Fig. 1J) and noxious prods and pinch produced graded firing of LSN neurons to different intensities of stimulation. Radiant heat stimulation selectively activating cutaneous layers of the skin failed to evoke activity of LSN neurons, suggesting that the observed firing of LSN neurons was selective to deep tissue stimulation.

We used hypertonic saline to induce acute muscle pain (Alessandri‐Haber et al. 2005; Svendsen et al. 2005; Lund et al. 2010; Lei et al. 2014, 2015). Following administration of hypertonic saline to the flexor digitorum brevis of the hind paw, we were able to quantify a time‐dependent increase in the evoked activity of LSN neurons to mechanical and thermal stimulation lasting at least 1 h (Fig. 1A–I). The peak effect shown in Figure 1J and K demonstrate a sensitization of LSN neurons to deep tissue mechanical stimulation and the development of cutaneous sensitivity over a large receptive field as illustrated by the novel firing to brush stimulation (Fig. 1J: 26 g vF *P* < 0.05, 60 g vF *P* < 0.001; Figure 1K: brush *P* < 0.05, prod 200 g/cm^2^ *P* < 0.05, prod 800 g/cm^2^ *P* < 0.001, pinch *P* < 0.001; all two‐way repeated measures ANOVA with Bonferroni post hoc tests).

**Figure 2:**
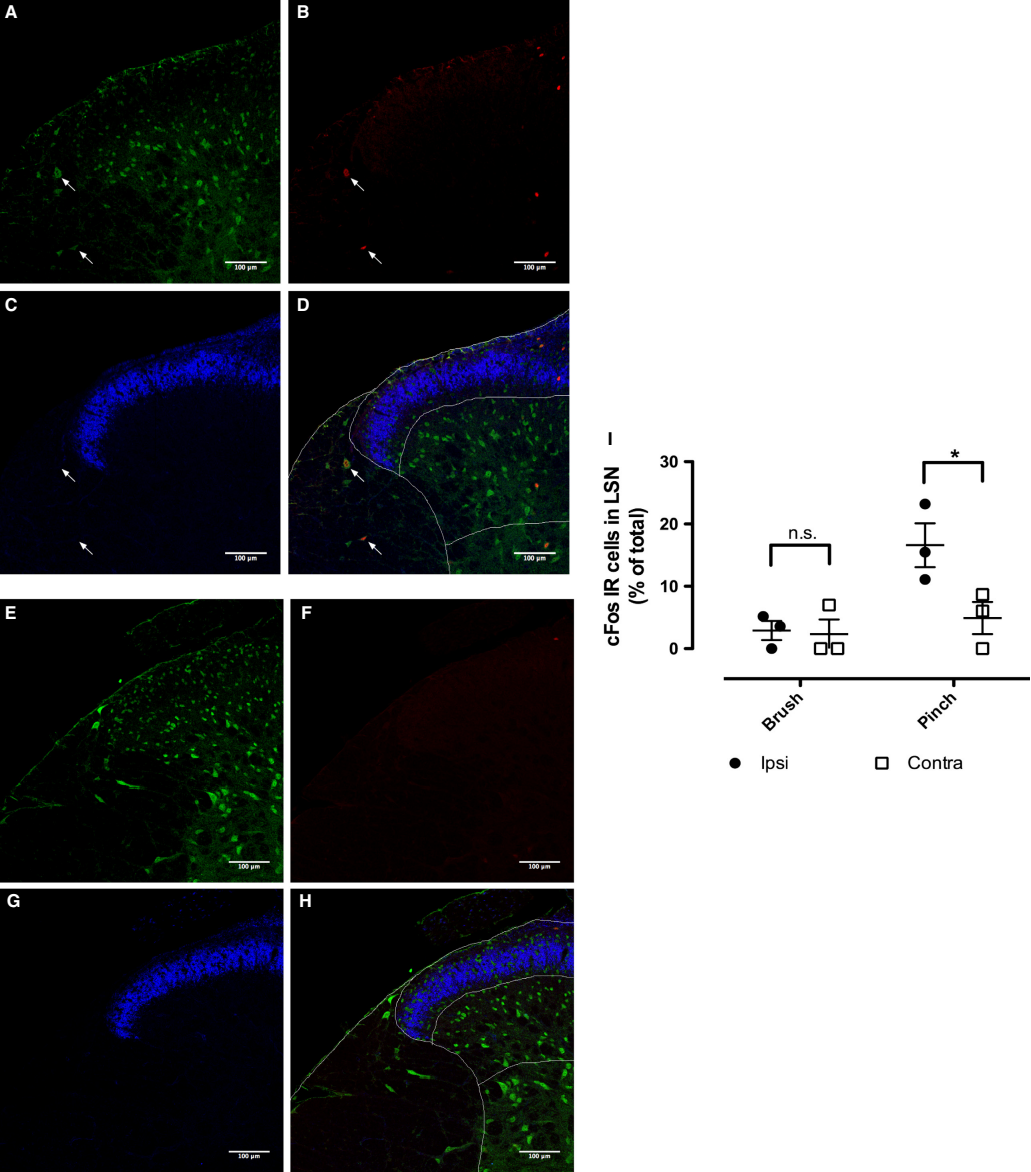
Immunohistochemical analysis of LSN activation following deep tissue stimulation. Dorsal horn sections immunostained for Fos (red), NeuN (green), and IB4 (blue) following pinch (A–D) and brush (E–H) stimulation of the hind paw. (D and H) Merged channels with superficial laminae, deep laminae, and LSN regions delineated. (I) Fos quantification in the LSN (*n* = 3 per group, two‐way repeated measures ANOVA with Bonferroni post hoc tests, *P < 0.05).

Immunohistochemical analysis of LSN Fos neural activation (Fig. 2) demonstrates a selective response to deep tissue stimulation (pinch) compared to cutaneous stimulation (brush). Example images in Figure 2A–D of pinch stimulated ipsilateral dorsal horn indicate Fos labeling in the LSN and superficial dorsal horn. Example images in Figure 2E–H of brush stimulated ipsilateral dorsal horn show insignificant labeling of Fos in the LSN or superficial dorsal horn. Comparing the percentage of Fos‐labeled cells in ipsilateral and contralateral LSN across both pinch and brush stimulated tissue reveals a significantly higher level of Fos labeling in the ipsilateral LSN after pinch stimuli when compared to all other treatment groups (Fig. 2I: two‐way repeated measures ANOVA with Bonferonni post hoc tests).

### Primary afferent input to the lateral spinal nucleus

To determine if the LSN receives any direct input from primary afferent fibers, we used an Advillin‐EGFP mouse to measure sensory innervation. This transgenic mouse expresses GFP under the advillin promotor, a protein known to be specifically expressed in sensory neurons and absent from CNS neurons (Akopian and Wood 1995; Hasegawa et al. 2007; Lau et al. 2011).

**Figure 3:**
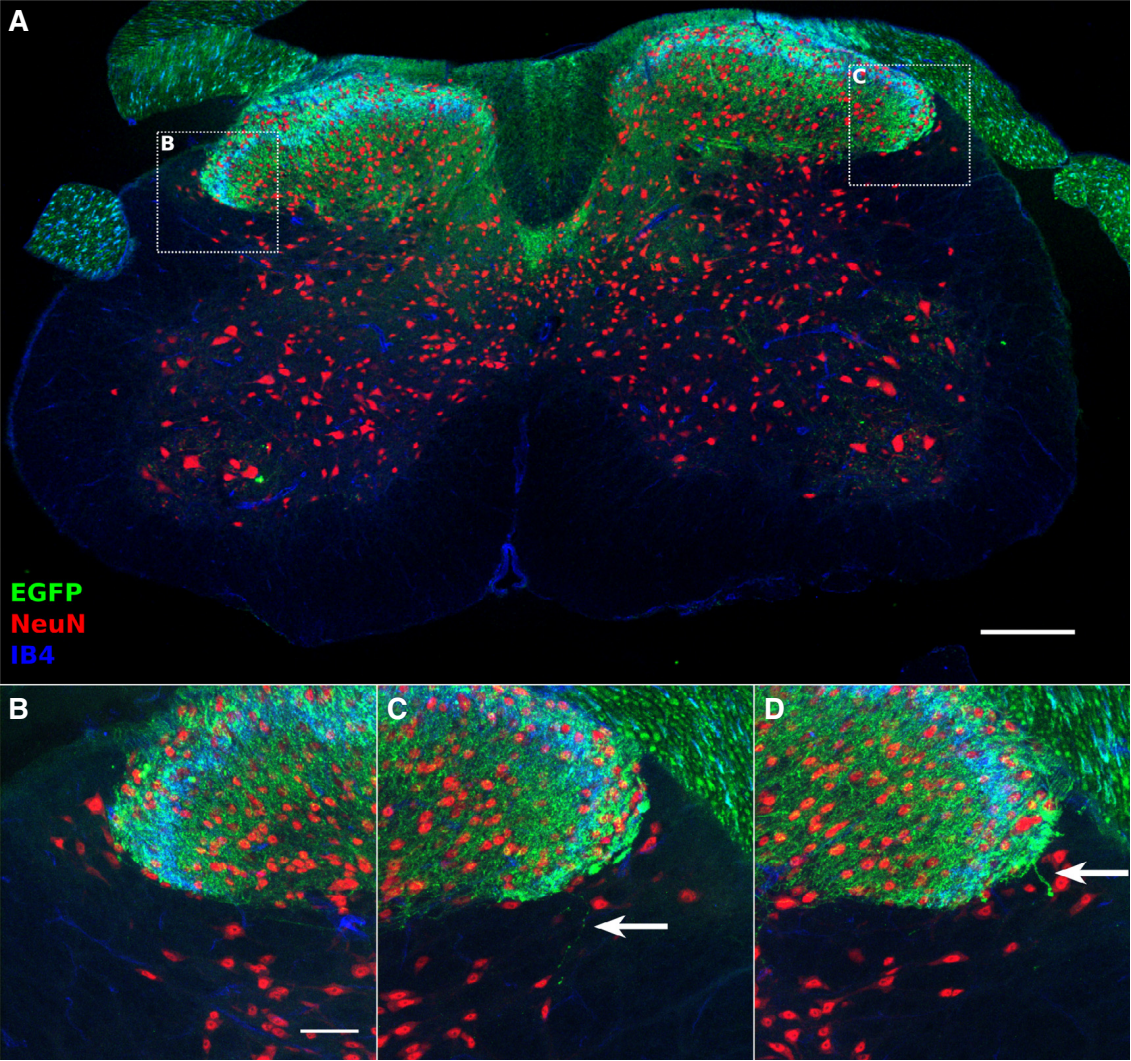
Primary afferent innervation of the LSN at the lumbar level. Transverse section of Advillin‐EGFP mouse spinal cord immunostained for NeuN (red) and EGFP (green) and IB4 (blue) (A). An example of a projected LSN image receiving no EGFP‐positive axonal inputs (B), or showing sparse inputs from EGFP‐positive axons (C, D). Arrows indicate EGFP‐positive axons entering the LSN territory. Scale bar: 200 *μ*m (A), 50 *μ*m (B).

Figure 3A shows a typical transverse spinal cord section from the Advillin‐EGFP transgenic mouse, with fluorescent labeling of GFP, NeuN, and IB4. GFP expression is consistent with primary afferent expression, showing localization to the dorsal columns, the dorsal roots, sparse input into deeper dorsal horn and the ventral horn, and a large arborization within the superficial dorsal horn. Figure 3B shows an example of a noninnervated LSN. The GFP signal shows a strong localization to the dorsal horn gray matter and does not cross into the dorsolateral funiculus. This innervation pattern was seen in 66% (29/44 images) of LSN samples.

Insets of the LSN innervation by EGFP‐positive axons are shown in Figures 3C and 3D. Very sparse EGFP‐positive axons are seen in LSN sample projections, often consisting of a single axonal arbor, as indicated by arrows. This pattern of very sparse innervation was observed in 34% (15/44 images) of LSN samples assessed. Thus, the LSN receives very little innervation from primary afferent fibers in the lumbar spinal cord.

### Neurons in the thalamic ventrobasal complex mediate cutaneous and deep tissue nociception

**Figure 4:**
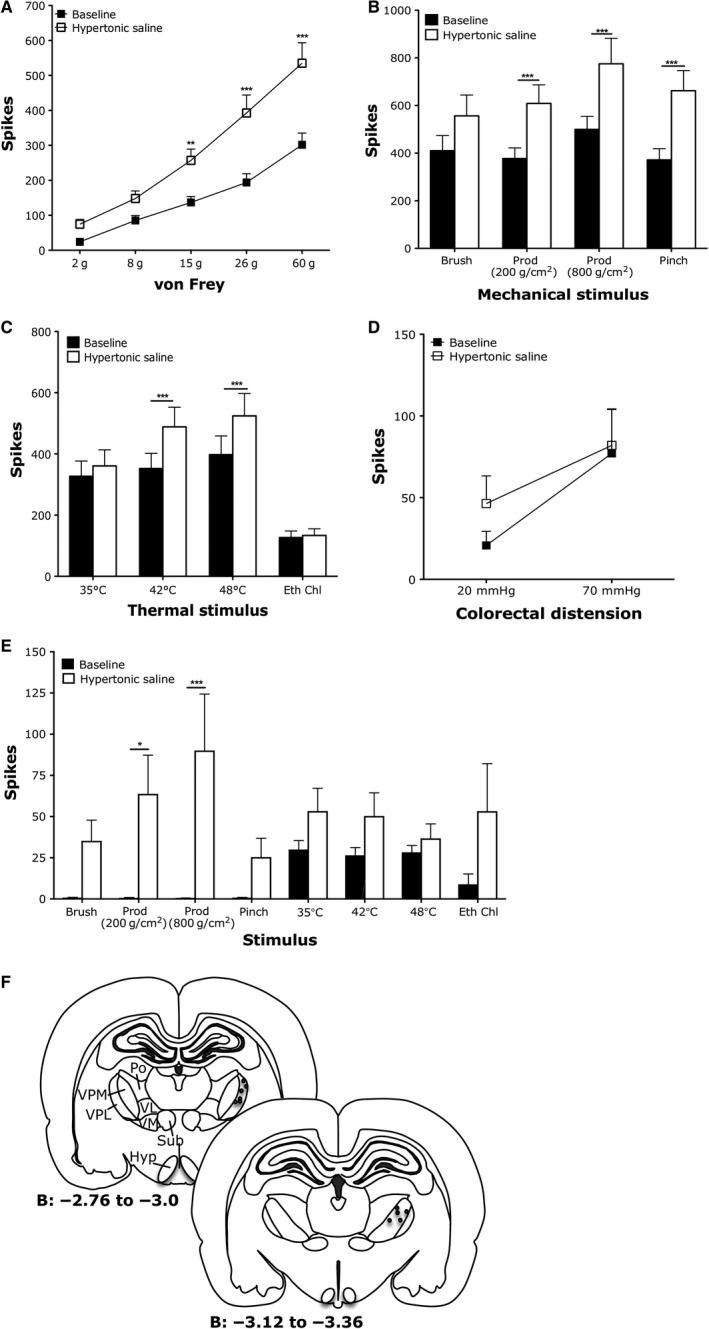
Polymodal and thermospecific neurons in the thalamic ventrobasal complex are sensitized to mechanical and thermal stimulation following intramuscular hypertonic saline. (A) Graded coding of polymodal thalamic neurons to punctate mechanical vF stimulation and increased firing to high intensity stimulation following i.m. hypertonic saline. (B) Increased firing of polymodal thalamic neurons to noxious mechanical and (C) noxious thermal stimulation following i.m. hypertonic saline. (D) Evoked firing of polymodal neurons to visceral stimulation. (E) Sensitization of thermospecific neurons to deep tissue mechanical stimulation following i.m. hypertonic saline. (F) Recording sites (*n* = 33 neurons for A–C, *n* = 14 for (E), all two‐way ANOVA with repeated measures, *P < 0.05, *P < 0.01, ***P < 0.001).

Neurons recorded in the VBC (Fig. 4F) were classified as polymodal (responding to innocuous and noxious intensities of mechanical and thermal stimulation) or thermospecific (responding only to thermal stimulation). We were unable to class any VBC neurons as selectively responsive to deep tissue stimulation, as even nociceptive‐specific neurons also responded to brush stimulation in a manner similar to lamina I dorsal horn neurons (data not shown) (Sikandar et al. 2013). Firing of polymodal VBC neurons was elicited by punctate mechanical vF stimulation in an intensity‐dependent fashion (Fig. 4A), as well as non‐noxious brush stimulation and noxious prods and pinch (Fig. 4B). Polymodal VBC neurons also responded to heat, noxious cold (ethyl chloride) (Fig. 4C), and visceral stimulation (Fig. 4D) in a graded fashion. Following intramuscular hypertonic saline, polymodal VBC neurons were sensitized to both mechanical and thermal, but not visceral, stimulation (Fig. 4A–D). Evoked activity to noxious punctate stimulation was enhanced (Fig. 4A; 15 g vF *P* < 0.01, 26 g vF *P* < 0.001, 60 g vF *P* < 0.001; two‐way repeated measures ANOVA with Bonferroni post hoc tests). Evoked activity to noxious prod and pinch stimulation, but not innocuous brush, was also augmented with hypertonic saline (Fig. 4B: prod 200 g/cm^2^ *P* < 0.001, prod 800 g/cm^2^ *P* < 0.001, pinch *P* < 0.001; two‐way repeated measures ANOVA with Bonferroni post hoc tests). Furthermore, evoked activity to noxious heat was enhanced with hypertonic saline, but not noxious cold responses (Fig. 4C: 42°C *P* < 0.001, 48°C *P* < 0.001; two‐way repeated measures ANOVA with Bonferroni post hoc tests).

Notably the evoked firing of thermospecific VBC neurons by thermal stimulation is less than observed for polymodal VBC neurons (Fig. 4E). Following intramuscular hypertonic saline, thermospecific VBC neurons were sensitized significantly to noxious prod and pinch mechanical stimulations (Fig. 4E: prod 200 g/cm^2^ *P* < 0.05, prod 800 g/cm^2^ *P* < 0.001; two‐way repeated measures ANOVA with Bonferroni post hoc tests), with a new response profile (although not statistically significant) to both innocuous brush and pinch. Evoked firing to thermal stimulation was not significantly different from baseline, although there was a trend toward enhanced firing following hypertonic saline administration.

## Discussion

This study presents two main findings. First, we used electrophysiology and immunohistochemistry to demonstrate a role for the lateral spinal nucleus in mediating deep tissue nociception in rats and confirm that direct primary afferent projection to the LSN is sparse (and in many cases absent). Second, we found that neural coding of higher order neurons in the thalamic ventrobasal complex is not selective for deep tissue nociceptive processing, although the polymodal and thermospecific subpopulations of VBC neurons can be sensitized to somatosensory inputs in an acute model of muscle pain.

The primary finding of a spinal nucleus that mediates deep tissue nociception provides crucial differentiation between the sensory processing of cutaneous inputs and muscle inputs within the central nervous system. Indeed the discrepancy in perceived muscle and cutaneous pain suggests differences in the peripheral and/or central processing of these inputs (Svensson et al. 1997). Our findings support earlier reports that the LSN can be activated by primary afferent input as LSN neurons, like those of lamina I, receive both monosynaptic and polysynaptic A*δ* and C fiber input (Ling et al. 2003; Pinto et al. 2010).

The LSN has previously been shown to receive sparse primary afferent input from visceral afferents via the lateral collateral pathway in rodent, likely from small to medium diameter primary afferent fibers, which may directly contribute to LSN activity following deep tissue stimulation (Neuhuber 1982; Neuhuber et al. 1986). Our data support these findings, as we have observed a very sparse input to the LSN from labeled primary afferent fibers. However, given the low level of this innervation, as well as its reported selectivity to visceral nerves, the input observed from deep cutaneous stimuli in the LSN this study is likely to be derived from polysynaptic inputs from the dorsal horn gray matter.

A comparison of evoked activity of LSN neurons to the more frequently characterized dorsal horn neurons may provide better understanding of the differences in spinal sensory discrimination and neural coding of skin and deeper inputs. We observed a clear, graded coding of LSN neurons to increasing intensity of mechanical stimulation that emulated our previous reports of neural coding of dorsal horn neurons (Sikandar et al. 2013). However, LSN neurons exhibit notably reduced firing frequency compared to dorsal horn excitability to similar intensities of mechanical stimulation (Sikandar et al. 2013), as well as a complete lack of firing to stimuli that were selectively activating cutaneous tissue, that is, dynamic brush and 8 g vF hair. The reduction in evoked firing of LSN neurons to peripheral stimulations compared to dorsal horn neurons may be due to the basal excitability of the nucleus itself from top‐down and spinal networks, or a smaller primary afferent convergence onto LSN neurons that results in less second‐order neuronal firing (Suzuki et al. 2002; Torsney and MacDermott 2006). In support of the electrophysiological data presented in this study, the number of c‐Fos‐positive neurons following pinch stimuli, but not brush stimuli, within the LSN were significantly elevated. Taken together, these data suggest the LSN responds selectively to deep tissue afferent stimulation preferentially over cutaneous stimulation, consistent with previous findings (Menetrey et al. 1980).

Intramuscular hypertonic saline is a widely used model to induce acute muscle pain in rodents (Alessandri‐Haber et al. 2005; Svendsen et al. 2005; Lund et al. 2010; Lei et al. 2014, 2015) and humans (Graven‐Nielsen et al. 1997a,b; Tsao et al. 2010). Injection of hypertonic saline into the gastrocnemius can produce a long‐lasting (several hours) secondary mechanical hypersensitivity of the hind paw (Lei et al. 2015). Indeed this infers the induction of central sensitization and expansion of central receptive fields (Sandkuhler 2009). The sensitization of primary afferents by hypertonic saline is thought to involve TRPV4 transduction, thereby suggesting that slight changes in osmolarity of primary afferent environments can influence the excitability of second‐order spinal cord neurons (Liedtke and Friedman 2003; Alessandri‐Haber et al. 2005). In our study, following administration of hypertonic saline to the flexor digitorum of the hind paw (underlying the receptive field for our LSN neuronal recordings), mechanically evoked activity of LSN neurons to mechanical stimulation of deep tissue was significantly enhanced. Furthermore, we observed a development of cutaneous sensitivity of LSN neurons, suggesting the induction of central changes to allow for the development of secondary hyperalgesia and spread of receptive fields to extend from muscle tissue to overlying cutaneous sites. It is possible that collaterals between the superficial dorsal horn (receiving cutaneous as well as muscle afferents) and the LSN permitted referral of LSN neuronal receptive fields to skin sites (Menétrey and Besson 1981).

Our observation that radiant heat, which selectively activates skin nociceptors, does not evoke LSN neuronal activity supports the notion that the LSN only mediates deep tissue sensory processing under normal conditions. Selective thermal activation of muscle primary afferents would be necessary to ascertain the neural coding of LSN neurons to heat stimuli, given earlier evidence of the distinct differences in the transduction and central processing of different modalities in normal and pathological deep pain states (Rau et al. 2007) (Sluka 2002). Different rates of skin heating have been reported previously to influence Fos immunoreactivity in the LSN (Koutsikou et al. 2007), although absolute cutoff temperatures of the heat stimuli and peak temperatures of stimulated skin were higher than the radiant stimulation used in this study (McMullan and Lumb 2006; Koutsikou et al. 2007; Sikandar et al. 2013).

The VBC includes the ventroposterolateral and ventroposteromedial nuclei that receive low‐threshold mechanosensory input from dorsal column nuclei and nociceptive inputs from dorsal horn projections in a somatotopic fashion (Iwata et al. 1992; Gauriau and Bernard 2004). Like previous studies, we found both polymodal and thermospecific neurons in the VBC that responded to somatosensory and visceral stimuli of increasing intensities in a graded fashion (Guilbaud et al. 1980; Peschanski et al. 1980; Sherman et al. 1997; Al‐Chaer et al. 1998; Han et al. 1998). Although we came across high‐threshold mechanoresponsive neurons (data not shown), we were unable to identify a subpopulation of neurons responding specifically to deep somatic stimulation comparable to our findings in the LSN. This may indicate a convergence of somatosensory inputs at the supraspinal level (Harris 1978; Monconduit et al. 2003), also supported by primate studies that demonstrate the convergence of cutaneous and muscle inputs onto spinothalamic tract neurons (Foreman et al. 1977, 1979a,b). On the other hand, our findings may be subject to a lack of sensitivity of the recording technique to obtain inputs from the relatively small receptive field of the digitorum flexor muscle compared to larger hind limb flexor muscles (Craig and Kniffki 1985; Kawakita et al. 1993). Under pathological conditions of intramuscular hypertonic saline, the evoked activity of polymodal neurons was sensitized to noxious somatosensory stimuli, Surprisingly, we also found that thermospecific neurons underwent a phenotypic switch; following intramuscular hypertonic saline, these neurons respond to innocuous and noxious mechanosensory inputs. Along with previous reports of injury‐induced changes in the activation thresholds and receptive fields of mechanosensory (Sherman et al. 1997) and polymodal (Patel and Dickenson 2016) VBC neurons, our data suggest that muscle trauma can alter thalamic processing of modality‐specific somatosensory inputs. It is conceivable that injury‐induced changes in spatiotemporal patterns of thalamic activity to somatosensory inputs would also alter perceived algosity and unpleasantness of nonpainful and painful inputs (Fields 1999; Price 2000).

In summary, the findings of our study demonstrate a role for the lateral spinal nucleus in mediating deep tissue nociception, despite sparse innervation by primary afferent fibers. In contrast, we did not find a subpopulation of thalamic neurons with a distinct response profile for processing deep somatic nociceptive inputs. However, we showed that acute muscle inflammation was able to induce threshold‐ and modality‐specific phenotypic switching of polymodal and thermospecific thalamic neurons, respectively. Our findings at both the spinal (LSN) and supraspinal (VBC) levels suggest the convergence of cutaneous and deep somatosensory inputs onto spinothalamic pathways, which are unmasked by activation of muscle nociceptors and consequent phenotypic alterations in LSN and VBC neural coding of somatosensory stimulation. Further studies, potentially using primate tissue, could verify the evolutionary conservation of the lateral spinal nucleus and the anatomical basis of cutaneous and deep somatosensory convergence at both the spinal and thalamic levels.

## Conflict of Interest

None declared.
